# Retinoblastoma Protein Paralogs and Tumor Suppression

**DOI:** 10.3389/fgene.2022.818719

**Published:** 2022-03-18

**Authors:** Mauricio Flores, David W. Goodrich

**Affiliations:** Roswell Park Comprehensive Cancer Center, Department of Pharmacology and Therapeutics, Buffalo, NY, United States

**Keywords:** tumor suppressor gene, pocket protein, retinoblastoma, cell cycle, cellular differentiation, therapeutic resistance

## Abstract

The retinoblastoma susceptibility gene (*RB1*) is the first tumor suppressor gene discovered and a prototype for understanding regulatory networks that function in opposition to oncogenic stimuli. More than 3 decades of research has firmly established a widespread and prominent role for *RB1* in human cancer. Yet, this gene encodes but one of three structurally and functionally related proteins that comprise the pocket protein family. A central question in the field is whether the additional genes in this family, *RBL1* and *RBL2*, are important tumor suppressor genes. If so, how does their tumor suppressor activity overlap or differ from *RB1*. Here we revisit these questions by reviewing relevant data from human cancer genome sequencing studies that have been rapidly accumulating in recent years as well as pertinent functional studies in genetically engineered mice. We conclude that *RBL1* and *RBL2* do have important tumor suppressor activity in some contexts, but *RB1* remains the dominant tumor suppressor in the family. Given their similarities, we speculate on why *RB1* tumor suppressor activity is unique.

## Introduction

The *RB1* tumor suppressor gene, discovered and isolated more than 30 years ago (Friend et al., 1986; Fung et al., 1987; Lee et al., 1987), has been the subject of extensive study due to its prominent role in cancer. Mutational loss of *RB1* function is the primary cause of the pediatric cancer retinoblastoma. Retinoblastoma either clusters in families as a hereditary susceptibility or it can arise sporadically. As predicted by the “two-hit hypothesis (Knudson, 1971; Comings, 1973),” hereditary retinoblastoma patients inherit a mutationally inactivated *RB1* allele from one parent while the remaining allele is inactivated somatically. Sporadic retinoblastoma, on the other hand, is associated with somatic genetic inactivation of both *RB1* alleles with the lower probability of two genetic hits accounting for the delayed age at diagnosis for these cases. DNA sequencing of retinoblastoma tumors has demonstrated few, if any, additional genetic alterations beyond those in the *RB1* gene (Zhang et al., 2012). This establishes *RB1* as a rare example where mutation of a single human gene is sufficient, or at least rate limiting, to cause a human cancer. *RB1* loss of function is involved in the development of other cancers as well. Hereditary retinoblastoma patients, for example, have increased risk of subsequent unrelated cancers (Schonfeld et al., 2021), indicating that mutational inactivation of *RB1* contributes to tumorigenesis in tissues beyond the retina. Cancer genome sequencing studies have confirmed *RB1* is genetically altered in a significant fraction of cases for many common adult cancers (see below). Experimental studies of mice genetically engineered to delete murine *Rb1* have confirmed that its loss, often in conjunction with other gene deletions, drives tumorigenesis in multiple tissues (Jacks et al., 1992; Meuwissen et al., 2003; Chen et al., 2004; MacPherson et al., 2004; Zhang et al., 2004; Zhou et al., 2006; Berman et al., 2008).

Soon after the molecular cloning of *RB1*, it became apparent there are two additional mammalian genes that it shares significant DNA sequence homology with (Ewen et al., 1991; Cobrinik et al., 1993; Hannon et al., 1993). These genes are now named *RBL1* (retinoblastoma-like 1) and *RBL2* (retinoblastoma-like 2) and their encoded proteins p107 and p130, respectively. Given their structural similarity to *RB1*, a central question has been whether *RBL1* and *RBL2* also function as tumor suppressor genes (Wirt and Sage, 2010; Indovina et al., 2013). It has been some years since published evidence relevant to this question has been reviewed, prior to widespread accumulation of human cancer genome sequencing data. The goal of this review is to re-examine evidence relevant to the tumor suppressor activity of the retinoblastoma protein paralogues, with an emphasis on recent DNA sequencing data from human cancer clinical specimens and experimental studies in genetically engineered mice. This evidence supports the hypothesis that all the paralogues can exhibit tumor suppressor activity in some biological contexts. However, the data also highlights a unique and more prominent role for *RB1*. We will highlight the diversity of retinoblastoma protein paralogue tumor suppressor activity and speculate on why pRb has unique tumor suppressor activity despite its structural and functional similarities to p107 and p130.

### Similarities in the Structure of Pocket Protein Paralogues

The pRb, p107, and p130 proteins are structurally related. The amino acid sequences most similar between them comprise a structural domain within the carboxy half of the proteins called the “pocket.” This pocket domain is the distinguishing feature of the family and is required for many of their known cellular and molecular functions. Indeed, human germline mutations conferring susceptibility to retinoblastoma disrupt this pocket structure. The pocket domain is also the most evolutionarily conserved feature among pRb-like orthologues across different species. Pocket proteins have been identified based on structural similarity and studied in diverse species across many eukaryotic phyla, including animals and plants. Functionally analogous proteins, although not structurally similar, have also been identified in yeasts (Hasan et al., 2013). Accompanying contributions to this research topic discuss pocket proteins in some of these other species.

The pocket domain is composed of two highly structured sub-domains separated by a less structured spacer. The structures of the subdomains have been solved by x-ray crystallography (Kim and Cho, 1997; Lee et al., 1998; Kim et al., 2001; Lee et al., 2002; Xiao et al., 2003; Liu and Marmorstein, 2007; Guiley et al., 2015) and both are reminiscent of cyclin box folds. There is also evidence that the amino half of pRb has a pocket-like structure composed of dual cyclin folds (Hassler et al., 2007). Consistent with cyclin folds in other proteins, the pocket protein cyclin folds provide surfaces that mediate key protein-protein interactions important for pocket protein function. Within the pocket domain itself, similarity between p107 and p130 (47% amino acid identity) is greater than between pRb and either p107 or p130 (<21% identity). The multiple dual cyclin folds with pocket proteins provides potential for multiple simultaneous protein interactions. While the pocket is the defining feature of these paralogues, it is important to note that both structured and unstructured regions outside the pocket also make important contributions to intra- and inter-molecular protein interactions and their regulation. In particular, the less structured carboxy terminal tail of pocket proteins also participates in important protein interactions, and the structure of this region is more distinct between pocket proteins (Rubin et al., 2005; Hirschi et al., 2010; Liban et al., 2017). Amino acid sequence divergence in protein regions outside of the pocket domains, including intrinsically disordered regions like the carboxy terminal tail, provide potential for functional diversity among the paralogues.

### Similarities in the Function of Pocket Protein Paralogues

All pocket proteins exhibit a predominant nuclear localization, although they have been detected in other cellular compartments as well (Hilgendorf et al., 2013). The multiple protein interaction surfaces present coupled with the absence of demonstrated enzymatic or sequence specific DNA/RNA binding activity suggest pocket proteins function as molecular adaptors mediating protein complex assembly. This hypothesis is consistent with the ability of pocket proteins to interact with a large variety of cellular and viral proteins, a topic reviewed elsewhere (Morris and Dyson, 2001; Goodrich, 2006; Chinnam and Goodrich, 2011; Dyson, 2016). Hundreds of pRb protein interactions have been identified, although only a subset have been well validated. The proteins interacting with p107 and p130 have not been as thoroughly characterized, yet it is evident that the cellular proteome capable of interacting with each of the three paralogues are only partially overlapping. Thus the binding affinity of a given cellular protein for pocket protein paralogues can differ significantly. A key example is the E2F family of sequence specific DNA binding transcription factors. Interaction with E2F transcription factors is the canonical pocket protein function presumed to mediate most of their important cellular effects like cell cycle control (see below). E2F1-3 have a binding preference for pRb while E2F4-5 have a binding preference for p107 and p130. Pocket protein paralogues are thus localized to specific regions of the genome based on the DNA binding specificity of the transcription factors with which they physically interact. In turn, the pocket proteins recruit chromatin regulatory complexes to these genome locations thus influencing gene expression. With some exceptions, the pocket proteins recruit chromatin regulatory complexes that suppress RNA transcription from nearby genes. The partially overlapping protein interactions between paralogues is also reflected in the distinct transcriptional regulatory complexes they participate in and the resulting transcriptional outputs (Black et al., 2003; Litovchick et al., 2007; Pilkinton et al., 2007).

The canonical cellular function of pocket proteins is to negatively regulate the cell division cycle (Goodrich et al., 1991; Zhu et al., 1993; Claudio et al., 1994). It is of interest that the pocket proteins are differentially expressed throughout the cell cycle. *RBL2* is most highly expressed in non-proliferating cells, quiescent or differentiated for example. *RBL1* expression is highest in proliferating cells as cells enter S phase. *RB1* expression is more uniform, expressed in both non-proliferating and proliferating cells throughout the cell cycle. E2F binding sites exist near many genes critical for the cell cycle, and pocket protein mediated silencing of RNA transcription from these genes enforces cell cycle regulation. The differential timing of pocket protein expression suggests they likely cooperate to enforce cell cycle control in different biological contexts. The broader expression of *RB1* throughout the cell cycle foreshadows its broader impact on cancer. It has also been noted that *RB1* has additional cancer relevant non-canonical functions that likely contribute to its tumor suppressor activity (Dick et al., 2018; Knudsen et al., 2019), a topic to which we return below.

### Similarities in the Regulation of Pocket Protein Paralogues

**FIGURE 1:**
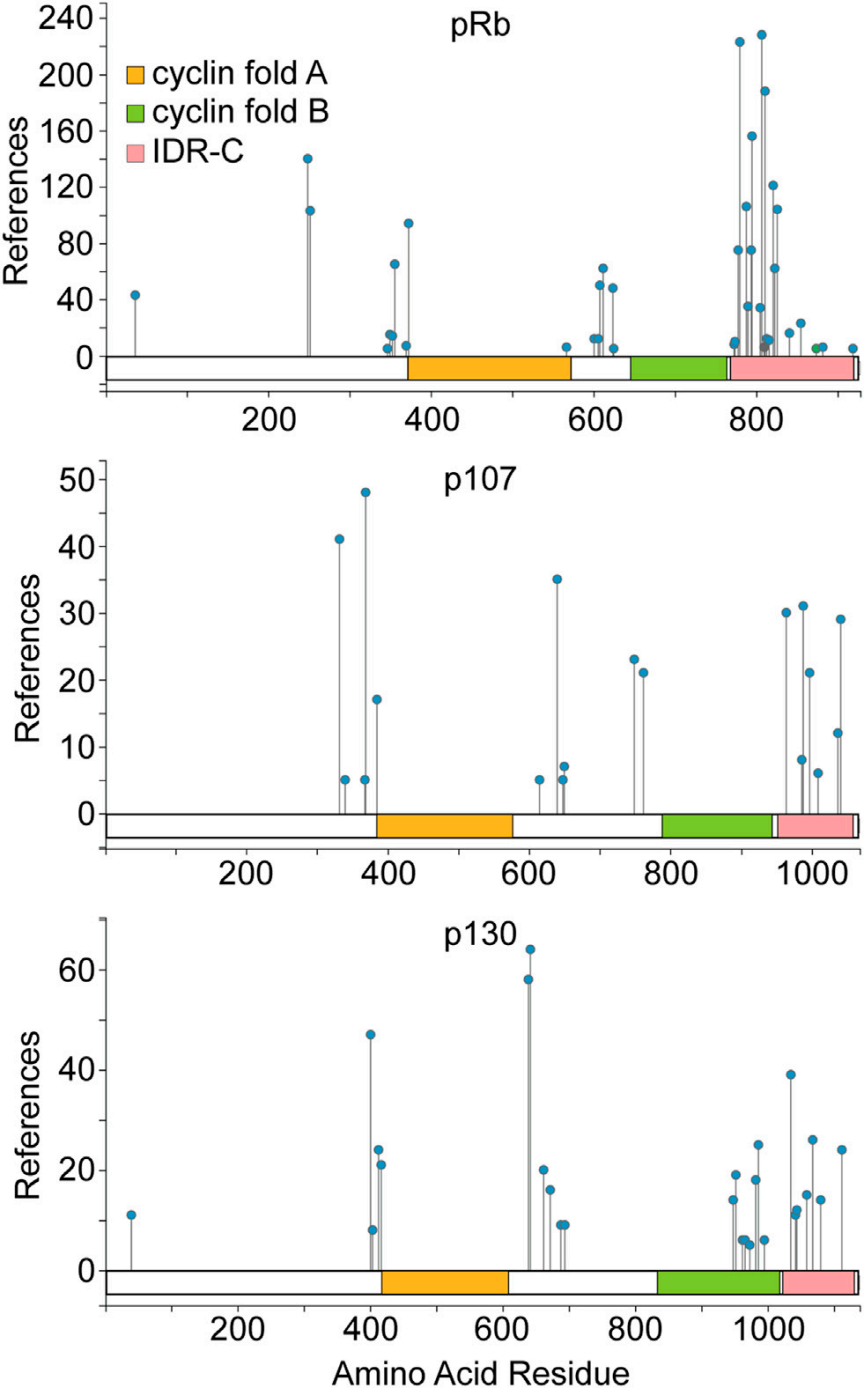
Pocket protein domain structure and regulation by phosphorylation. A schematic for each pocket protein paralogue is shown highlighting the amino acid residues comprising the dual cyclin fold pocket (indicated by orange and green shading) for each protein as well as the intrinsically disordered C terminal region. Pocket protein phosphorylation sites identified most commonly in the published literature are indicated, as compiled in the PhosphoSitePlus database from nearly 10,000 journal articles (Hornbeck et al., 2015). The number of independent studies within this compilation that identify a given site is plotted on the Y axis. Phosphorylation patterns of the pocket protein paralogues are broadly similar with phosphorylation sites clustering in less structured regions flanking the cyclin folds. The figure is adapted from PhosphoSitePlus.

Mitogenic signals normally activate kinases that phosphorylate the pocket proteins, cyclin dependent kinases (CDK) paramount among them. These phosphorylation events can relieve pocket protein mediated cell cycle suppression. Phosphorylation of pRb occurs on as many as 44 different amino acid residues (Hornbeck et al., 2015), although less than half of these are recurrently detected in the majority of phospho-proteome studies. The most commonly phosphorylated and evolutionarily conserved sites are proline directed, consistent with phosphorylation by CDKs or other proline directed kinases. All the pocket proteins exhibit an analogous overall phosphorylation pattern (Figure 1). Phosphorylation sites tend to cluster in the less structured regions of the proteins flanking the cyclin fold subdomains. Experimental evidence demonstrates that CDKs phosphorylate the pocket proteins directly and that this phosphorylation inhibits their cell cycle suppression activity (Connell-Crowley et al., 1997; Hansen et al., 2001; Leng et al., 2002; Tedesco et al., 2002). The structure of some phosphorylated pocket protein domains have been solved, identifying molecular interactions that regulate pocket protein function (Rubin et al., 2005; Burke et al., 2010; Hirschi et al., 2010; Burke et al., 2012). This regulation involves phosphorylation of less structured regions of the proteins that mediate conformational changes influencing both intra- and inter-molecular protein interactions. Intra-molecular interactions can regulate access to pocket contacts essential for E2F binding. Another structural model suggested by Liban et al. (2017) indicates phosphorylation interferes directly with inter-molecular contacts between the intrinsically unstructured carboxy terminus of the pocket proteins and E2F. This model also helps to explain how structural divergence in the carboxy terminal region among paralogues dictates binding preferences for specific E2F family members. Observations such as these confirm the notion that structural divergence between pocket protein paralogues can specify functional diversity.

**FIGURE 2:**
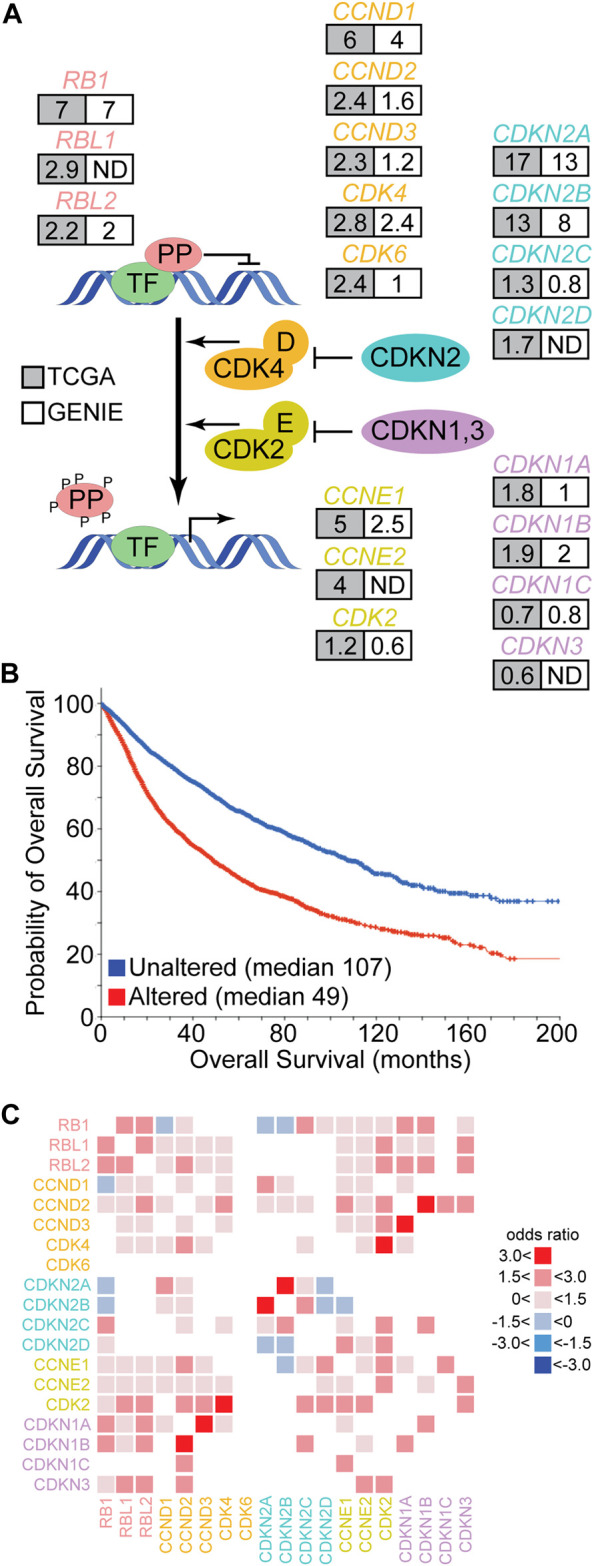
Genetic alteration of the pocket protein pathway in human cancer. **(A)** The schematic depicts the core pocket protein pathway and its regulation by CDK mediated phosphorylation. PP represent the pocket proteins and TF represent transcription factors like the E2F family. Each gene of the core PP pathway is listed with the frequency (%) of genetic alteration in cancer based on TCGA (grey) or GENIE (white) pan-cancer databases. ND indicates the gene was not measured. **(B)** Overall survival data collected by the TCGA was segregated into those cancers containing genetic alteration of at least one PP pathway member (red) or those cancers without alterations in the PP pathway (blue). The probability of overall survival is plotted over time with median survival times listed. Figure modified from cBioPortal (Cerami et al., 2012; Gao et al., 2013). **(C)** TCGA pan-cancer data was used to identify significant pairwise co-occurrence (red) or mutually exclusive (blue) interactions between PP pathway members within individual cancer samples (*q* < 0.05). The shade of red or blue is based on the odds ratio calculated for the given interaction.

Growth suppressive signals can maintain or induce pocket protein mediated cell cycle suppression by activating CDK inhibitory proteins (CDKi). There are at least eight proteins within this larger CDKi family, four encoded by the *CDKN2A-D* genes that target CDK4/6 enzymes preferentially and four encoded by *CDKN1A-C* plus the *CDKN3* gene that target CDK2 enzymes. CDKi, CDKs and the pocket proteins together comprise the core pocket protein cell cycle regulatory pathway (Figure 2A). Early evidence suggested a quantitative regulatory model for this pathway wherein the balance of CDK and CDKi activity dictates how extensively the pocket proteins are phosphorylated. Accumulation of pocket protein phosphorylation events beyond a threshold would disrupt pocket protein mediated protein interactions causing de-repression of cell cycle genes and stimulating cell cycle progression. This model has been based on the observation that pRb “hyperphosphorylation,” recognized by a shift in protein mobility during SDS-PAGE, correlates with the G1 to S cell cycle phase transition and that ectopic cyclin/CDK expression can drive pRb hyperphosphorylation and the cell cycle (Buchkovich et al., 1989; Chen et al., 1989; DeCaprio et al., 1989). However, CDKs have their own pocket protein phosphorylation site preferences. Sites preferred by the growth factor responsive CDK4/6 enzymes appear to be particularly important for initiating regulation of pocket protein cell cycle suppression (Connell-Crowley et al., 1997; Hansen et al., 2001; Leng et al., 2002; Tedesco et al., 2002; Schade et al., 2019), but cooperation with CDK2 enzymes is likely necessary for hyperphosphorylation (Hatakeyama et al., 1994; Weinberg, 1995).

More recent data has complicated this quantitative model by demonstrating that pRb is not progressively phosphorylated by CDK4 enzymes in G1 phase. Rather CDK4 appears to phosphorylate pRb once and only once at many different amino acid residues (Narasimha et al., 2014). This generates multiple monophosphorylated pRb isoforms that exhibit distinct protein interaction preferences and transcriptional outputs (Sanidas et al., 2019; Hattori et al., 2014). Once cyclin E/CDK2 is activated in late G1, pRb is more completely functionally inactivated by hyperphosphorylation. This is complicated further by the observation that other kinases can also phosphorylate pRb (Gubern et al., 2016; Inoue et al., 2007; Nair et al., 2009; Nath et al., 2003; Hou et al., 2002; Guo et al., 2005; Dasgupta et al., 2004; Ventura et al., 2018) and that additional pocket protein post-translational modification are apparent (Chan et al., 2001; Nguyen et al., 2004; Markham et al., 2006; Carr et al., 2011; Leduc et al., 2006; Cho et al., 2012; Meng et al., 2016; Ledl et al., 2005; Miwa et al., 2006; Kim et al., 2015; Saddic et al., 2010; Munro et al., 2010). It is thus increasingly clear that pRb is regulated both quantitatively and qualitatively. As p107 and p130 are not as extensively studied, it is not clear whether they are also monophosphorylated by CDK4/6 enzymes. However, it appears likely given the similarity in their overall phosphorylation patterns (Figure 1) (Hornbeck et al., 2015) and the observation that the mechanism of cyclin D CDK4/6 specific docking to pocket proteins is shared among the paralogues (Topacio et al., 2019).

In sum, these observations suggest the pocket protein paralogues share important structural, functional, and regulatory features. Yet each pocket protein paralogue diverges in these features as well, with p107 and p130 more similar to each other than either of them are to pRb. Given the interplay between post-translational modification, protein interactions, and pocket protein activity, pocket proteins are well situated to integrate output from cellular regulatory networks and translate them into changes in gene expression. Differences between pocket protein structure, regulation, and molecular interactions, therefore, likely account for their functional diversity.

### Pocket Protein Pathway Genetic Alterations in Primary Human Cancers

Human cancer genome sequencing studies demonstrate that the core pocket protein pathway is altered recurrently across a wide range of cancer types (Figure 2A) (Knudsen et al., 2020). For example, the Cancer Genome Atlas (TCGA) has molecularly characterized more than 10,000 patient samples across 32 cancer types. In this data set, the frequency of alteration for different pocket protein pathway genes ranges from 0.6 to 17% of cases (Gao et al., 2013). The GENIE pan-cancer database contains DNA sequence data from more than 110,000 patients and 100 cancer types. The frequency of genetic alteration for pocket protein pathway genes in this dataset ranges from 0.6 to 13% (Gao et al., 2013). Genetic alterations in the core pocket protein pathway are clinically significant as TCGA pan-cancer data indicates patients whose cancers exhibit a genetic alteration in at least one member of the pathway have an overall survival less than half that of patients whose cancer does not contain an alteration in the pathway (Figure 2B) (Gao et al., 2013). The genetic link between pocket protein pathway genetic alteration and cancer is strengthened further by data from hereditary retinoblastoma patients. These patients inherit one mutationally inactivated *RB1* allele and have increased risk for additional cancers unrelated to retinoblastoma later in life (Schonfeld et al., 2021). For example, hereditary retinoblastoma patients have significantly elevated risk of developing sarcomas. In the general population, pocket protein pathway alteration also correlates with reduced overall survival for patients suffering from sarcomas (log rank *p* < 0.05) (Table 1). However, the correlation between pathway alteration and shorter overall survival is not uniform across cancer types. Assuming these differences are not entirely due to bias in sample size, they suggest pocket protein pathway alterations have non-uniform, cancer context dependent effects.

**TABLE 1:** Pocket protein pathway gene alterations and shorter overall survival^a^.

Cancer type	N	Log rank *P*
Glioma	514	4.3 × 10^−11^
Mesothelioma	87	1.6 × 10^−6^
Kidney	348	3.2 × 10^−5^
Sarcoma	255	1.5 × 10^−4^
Lung	1,053	2.4 × 10^−3^
Adrenocortical carcinoma	92	6.9 × 10^−3^
Pancreatic	184	0.02
Head and Neck squamous carcinoma	523	0.03
Uterine	586	0.054
Cholangiocarcinoma	36	0.06

a The top ten cancers ranked by log rank *p* value of the correlation between pocket protein pathway genetic alteration and overall survival are shown. Data is from the Cancer Genome Atlas and log rank *p* values are calculated on cBioPortal (Gao et al., 2013).

The pattern of alterations among pocket protein pathway genes is also informative. Analysis of pairwise interactions indicate that genetic alterations in different pathway members tend to co-occur within samples rather than to exhibit mutual exclusivity (Figure 2C). The only significant mutually exclusive interactions detected in TCGA data are between *RB1* and *CDKN2A*/B or CCND1, between CDKN2D and *CDKN2A*/B, and between CCNE1 and CDKN2B. In most cases, therefore, genetic alteration of multiple pathway genes is predicted to cooperate in driving cancer development. In some cases, this prediction has been confirmed by experimental studies (see below). It is entirely unclear how this cooperation may occur, but two general and complementary explanations are possible. The simplest potential explanation is that multiple genetic alterations each contribute quantitatively to more complete pathway functional inactivation. Functional redundancies within the pathway, within the pocket proteins themselves for example, may require genetic alteration of multiple members for more complete functional inactivation. It is also possible that genetic alterations in different pathway members yield qualitatively distinct functional effects, and multiple alterations would be required to inactivate different aspects of tumor suppressor functions mediated by the pathway.

The most frequently mutated genes in the pathway are *CDKN2A* and CDKN2B. As CDKi represent the most proximal regulatory input into the core pathway, this could imply that broad deregulation of CDK4/6 activity and pocket protein phosphorylation is most critical for relieving tumor suppression. However, this hypothesis has important caveats. The high frequency of *CDKN2A* and CDKN2B alteration is likely biased by unique features of this genetic loci. *CDKN2A* and CDKN2B map adjacent to each other on chromosome 9, so copy number losses or structural alterations in this region often affect both genes (odds ratio >3.0, *q*-value < 0.001). Further, *CDKN2A* encodes two distinct proteins through use of alternative reading frames. One of these reading frames encodes the CDK4/6 CDKi p16 while the other encodes p14ARF. p14ARF is important for maintaining the stability and activity of p53, so genetic loss of *CDKN2A* function may be selected in cancer because it compromises both the pocket protein and p53 tumor suppressor pathways. There is evidence from clinical specimens indicating loss of these pathways synergize based on the observation that genetic alterations in *RB1* and *TP53* tend to co-occur in pan-cancer analysis (*q* < 0.001). There is also abundant experimental evidence indicating these pathways cooperate to control cancer relevant biology, with two examples including cellular senescence (Ben-Porath et al., 2005) and therapeutic resistance (Quintanal-Villalonga et al., 2020). Alteration of both pathways in mouse models often yield a cancer phenotype in situations where alteration of either pathway alone does not (Meuwissen et al., 2003; Zhou et al., 2006). Given the potential bias inherent in the *CDKN2A*/B locus, it remains unclear whether genetic inactivation of *CDKN2A*/B is the most efficient means to functionally inactivate pocket protein pathway activity (see below).

**FIGURE 3:**
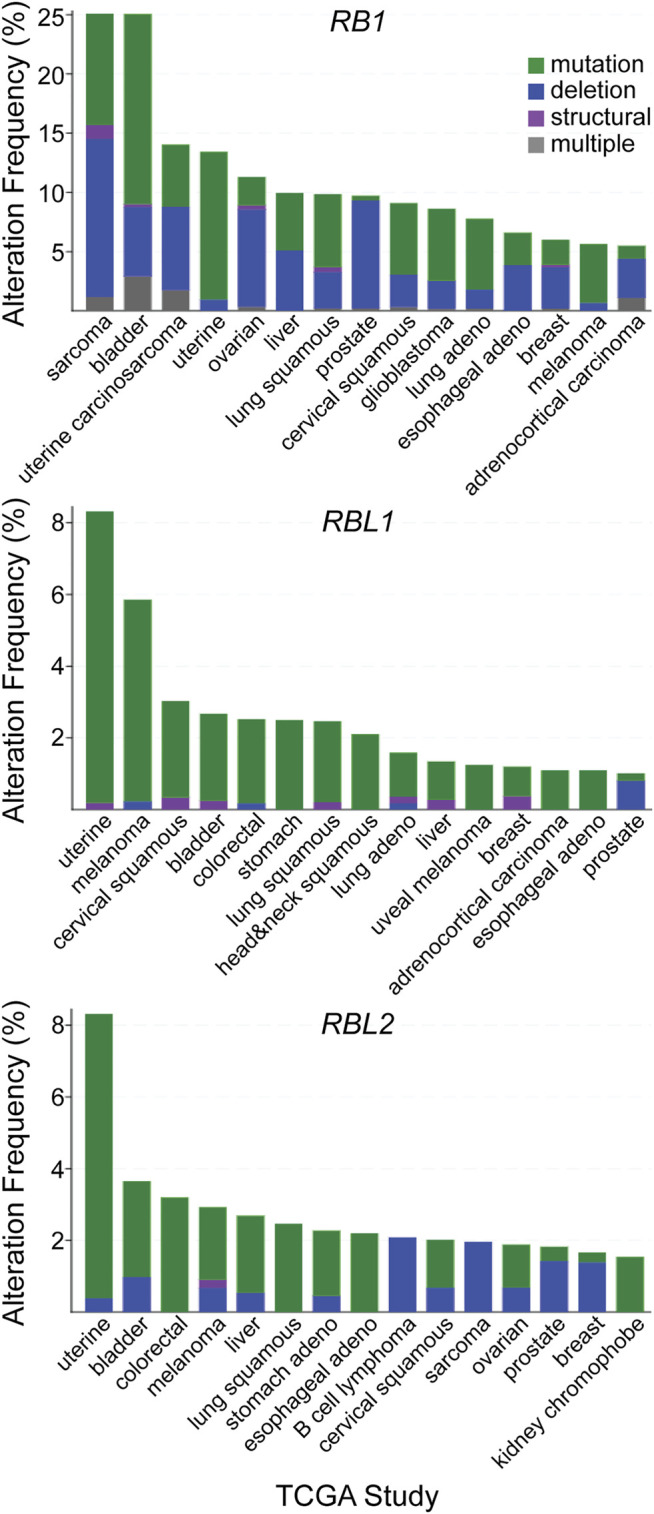
Patterns of pocket protein paralogue gene alteration in human cancer. The top fifteen TCGA cancer studies ranked by the frequency of debilitating genetic alterations observed for each individual pocket protein paralogue genes are listed. The nature of the debilitating genetic alteration is shown based on the indicated color code.

The frequency of *RB1* alteration (7%) is next highest among the remaining pathway genes, higher than the frequency of *RBL1* alteration (2.9%) or *RBL2* alteration (2.2%). Interestingly, alterations in *RBL1* and *RBL2* include a significant number of gene amplifications. While the impact of these amplifications on pathway activity is unclear, if we assume they do not compromise pocket protein function and exclude them from the analysis, then the frequency of debilitating genetic alterations for *RBL1* and *RBL2* alterations decline to 1.7% and 1.9% respectively. The top 15 cancer types ranked by frequency of debilitating mutation for each pocket protein gene is different (Figure 3). For most cancers the frequency of debilitating *RB1* alterations is greater than the frequency of *RBL1* or *RBL2* alterations. In some cancer types like sarcoma, this discrepancy is marked (*RB1* = 25%, *RBL1* = 0.4%, *RBL2* = 2%). In other cancers like melanoma, the frequency of pocket protein paralogue alterations is comparable (melanoma *RB1* = 5.6%, *RBL1* = 5.9%, *RBL2* = 3%). While the mutation frequency in *RB1* is typically greater than for *RBL1* or *RBL2*, there are some cancer subtypes where this is reversed. For example, mucinous adenocarcinomas of the colon exhibit few *RB1* alterations (1.6%) but more frequent *RBL1* (4.9%) or *RBL2* (8.2%) alterations, although case numbers are relatively low. It is also notable that the nature of the genetic alterations affecting the pocket proteins can vary by cancer type. For example, prostate cancer is characterized by pocket protein gene deletions while genetic alterations in uterine cancer are primarily single nucleotide mutations (Figure 3). This likely reflects the nature of genome instability prevalent in a given cancer type but may also indicate selection for different modes of pocket protein pathway inactivation. For example, the fraction of cancers exhibiting gene deletions versus other genetic alterations is generally higher for *RB1* than for *RBL1* or *RBL2*, suggesting selective pressure may favor complete functional inactivation of *RB1* alleles.

### Pocket Protein Pathway Genetic Alterations in Advanced Human Cancers

**FIGURE 4:**
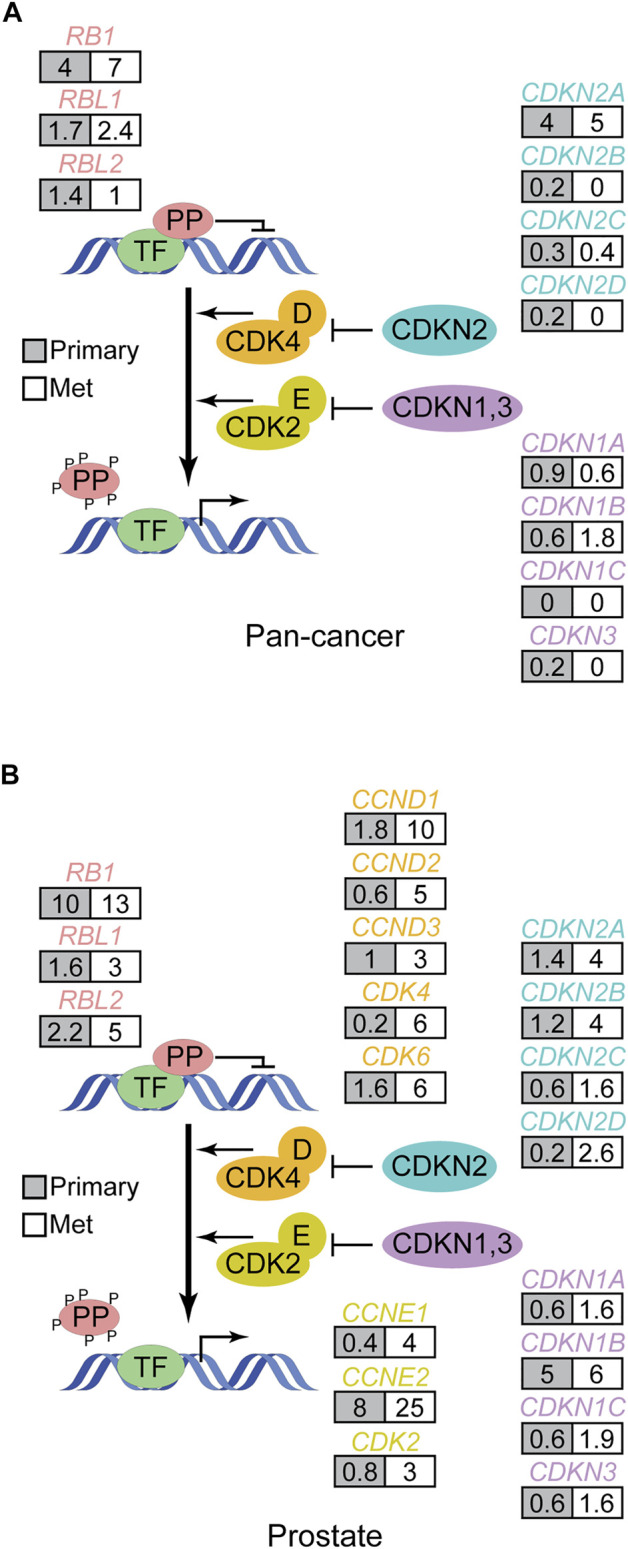
Pattern of pocket protein pathway gene alterations in metastatic human cancer. **(A)** The schematic in Figure 2 is modified to show how often (%) pocket protein pathway genes are genetically altered in pan-cancer genome wide DNA sequencing studies of metastatic solid cancers (Robinson et al., 2017; Priestley et al., 2019) compared to primary tumors (TCGA pan-cancer). Here grey boxes indicate primary tumors while white boxes reflect data from metastatic tumors. The data from metastatic cancers does not include copy number changes, so the analysis is restricted to debilitating mutations. **(B)** As in A but focused on primary and metastatic prostate cancer. Data is from Abida et al. (Abida et al., 2019) and TCGA and calculated in cBioPortal (Gao et al., 2013).

Most cancer genome sequencing data from solid cancers has been obtained from surgically resected primary tumors because of the relative difficulty in accessing patient specimens from more advanced stages of disease. However, studies analyzing clinical specimens from advanced cancers, like metastatic disease, are emerging. In the limited number of pan-cancer studies that have been performed on metastatic solid tumors to date, pocket protein genetic alterations are also recurrent (Priestley et al., 2019; Robinson et al., 2017). Indeed the pocket protein and *TP53* pathways appear to be two of the most frequently altered tumor suppressor pathways in metastatic solid cancers. The pattern of somatic pocket protein pathway mutations in metastatic cancers generally mimics that of primary tumors (Figure 4A). However, there are some interesting differences. As prostate cancers progress from primary tissue confined disease to metastatic disease, for example, there is a marked increase in the frequency of pocket protein pathway genetic mutations (Figure 4B). Metastatic prostate cancer can be treated successfully with androgen receptor signaling inhibitor therapy, although acquired therapeutic resistance is inevitable. One of the strongest genomic predictors of acquired therapeutic resistance and poor clinical outcome is *RB1* loss of function alterations (Abida et al., 2019). *RB1* status survives multivariate analysis as a significant predictor of time on therapy and overall survival. Thus pocket protein genetic alterations can occur late in prostate cancer progression, presumably to facilitate metastasis and acquired therapeutic resistance.

It is also becoming clear from such studies that *RB1* and *TP53* alterations are associated with prostate cancer lineage plasticity, transformation of prostate adenocarcinoma to an alternative lineage state no longer dependent on androgen receptor signaling. Neuroendocrine prostate cancer (NEPC) is one of the most obvious and common alternative lineage states that arise under pressure from androgen receptor signaling inhibitors, and this disease state arises by transdifferentiation from a pre-existing adenocarcinoma. The frequency of *RB1* alteration increases markedly in NEPC compared to advanced prostate adenocarcinoma (Table 2) (Abida et al., 2019; Laudato et al., 2019). In NEPC, alteration of *RB1* occurs more frequently than for alteration in any other core pocket protein pathway gene (Ku et al., 2017; Mu et al., 2017). It is noteworthy that alteration of the other pocket protein paralogues *RBL1* and *RBL2* is rare in both prostate adenocarcinoma and NEPC, suggesting loss of *RB1* has a unique role in cancer lineage plasticity in the prostate. The functional role of *RB1* alteration in NEPC transdifferentiation has been verified in experimental models of prostate cancer (Ku et al., 2017; Mu et al., 2017), but the potential role of the other paralogues has not been assessed.

**TABLE 2:** Pocket protein pathway gene alterations in advanced prostate cancer^a^

Gene	Neuroendocrine?	Alteration frequency (%)
***RB1***	**Yes**	**51**
	**No**	**8.1**
*RBL1*	Yes	0
	No	0
***RBL2***	**Yes**	**0**
	**No**	**0**
CCND1	Yes	7.3
	No	9.2
**CCND2**	**Yes**	**0**
	**No**	**0**
CCND3	Yes	7.3
	No	1.6
**CDK4**	**Yes**	**4.9**
	**No**	**4.9**
CDK6	Yes	7.3
	No	4.1
***CDKN2A***	**Yes**	**7.3**
	**No**	**1.9**
CDKN2B	Yes	2.4
	No	2.2
**CDKN2C**	**Yes**	**0**
	**No**	**1.1**
CDKN2D	Yes	0
	No	0
**CCNE1**	**Yes**	**12.2**
	**No**	**1.9**
CCNE2	Yes	0
	No	0
**CDK2**	**Yes**	**0**
	**No**	**0**
CDKN1A	Yes	0
	No	0
**CDKN1B**	**Yes**	**19.5**
	**No**	**2.4**
CDKN1C	Yes	0
	No	0
**CDKN3**	**Yes**	**0**
	**No**	**0**

^a^The frequency of pocket protein pathway gene alterations in advanced prostate cancer is shown, segregated by whether the cancers exhibit neuroendocrine features. Data is from Abida et al. (Abida et al., 2019) and calculated in cBioPortal (Gao et al., 2013). Bold text is added to improve legibility.

Analogous clinical observations have been made in other cancer types. Advanced EGFR mutant non-small cell lung cancers are now treated with EGFR tyrosine kinase inhibitors as standard of care. While very effective, nearly all patients will eventually relapse from this therapy. Alterations in the pocket protein pathway identify EGFR mutant lung cancer patients that have worse outcomes on this therapy (Niederst et al., 2015; Jiang et al., 2016; Blakely et al., 2017; Lee et al., 2017; Marcoux et al., 2019; Skoulidis and Heymach, 2019; Liu et al., 2021). Interestingly EGFR mutant lung cancer with concomitant *RB1*/*TP53* alterations are more likely to undergo neuroendocrine transformation to a small cell lung cancer-like disease, analogous to the neuroendocrine transdifferentiation observed in prostate cancer (Quintanal-Villalonga et al., 2020). In fact, neuroendocrine lineage variants can arise in many solid tumors beyond the two examples detailed here, and these neuroendocrine variants converge on a similar histological and molecular phenotype, including ubiquitious alterations in *RB1* and *TP53* (Balanis et al., 2019). *RBL1* and *RBL2* genetic alterations are not common in these neuroendocrine cancers, again indicating *RB1* alteration has a unique role in this cancer context.

### Pocket Protein Functional Activity in Cancers

An increasingly common approach for measuring pocket protein pathway functional activity is the use of gene expression signatures. These signatures are identified by comparing RNA transcription genome wide in experimental or clinical specimens with known pathway status, most commonly based on *RB1* status. The signatures are defined by comparisons in training sets and are then used to assess pathway activity by analysis of RNA-seq data from additional clinical or experimental specimens (Chen et al., 2019; Knudsen et al., 2020). As expected, these signatures correlate with genetic *RB1* inactivation, but they also identify samples with low pathway activity that retain wild type *RB1*. These expression-based measures of pathway activity predict cancer outcomes just like genetic alterations in the pocket protein pathway. Low pathway activity tends to correlate with worse clinical outcome. Comparison of gene expression signatures in samples with different alterations in the pathway suggest that pathway alterations other than *RB1* can yield transcriptional changes similar to those observed upon genetic *RB1* loss, primarily with respect to cell cycle regulatory genes. Yet it is also evident that functional suppression of pRb through alteration of upstream genes controlling CDK activity is not equivalent to genetic *RB1* inactivation as assessed by transcriptional output (McNair et al., 2018), and these differences generally affect the expression of genes not obviously relevant to the cell cycle.

DNA and RNA sequencing data from human cancer clinical specimens are consistent with the notion that all the pocket paralogues have tumor suppressor activity. However, the pattern of pocket protein genetic alterations among the paralogues is dependent on cancer type. Among paralogues, *RB1* loss of function alterations are more broadly observed across human cancer. In some contexts like neuroendocrine transdifferentiation, *RB1* loss is uniquely observed. The pattern of *RBL1* and *RBL2* genetic alteration across cancer types is generally more similar to each other than to *RB1* (Figure 3), consistent with structural similarities between the paralogues. This observational data is consistent with the notion that pocket protein paralogues have partially overlapping tumor suppressor functions, likely based on their overlapping role in cell cycle control, but that *RB1* has a more dominant and unique role in tumor suppression.

**FIGURE 5:**
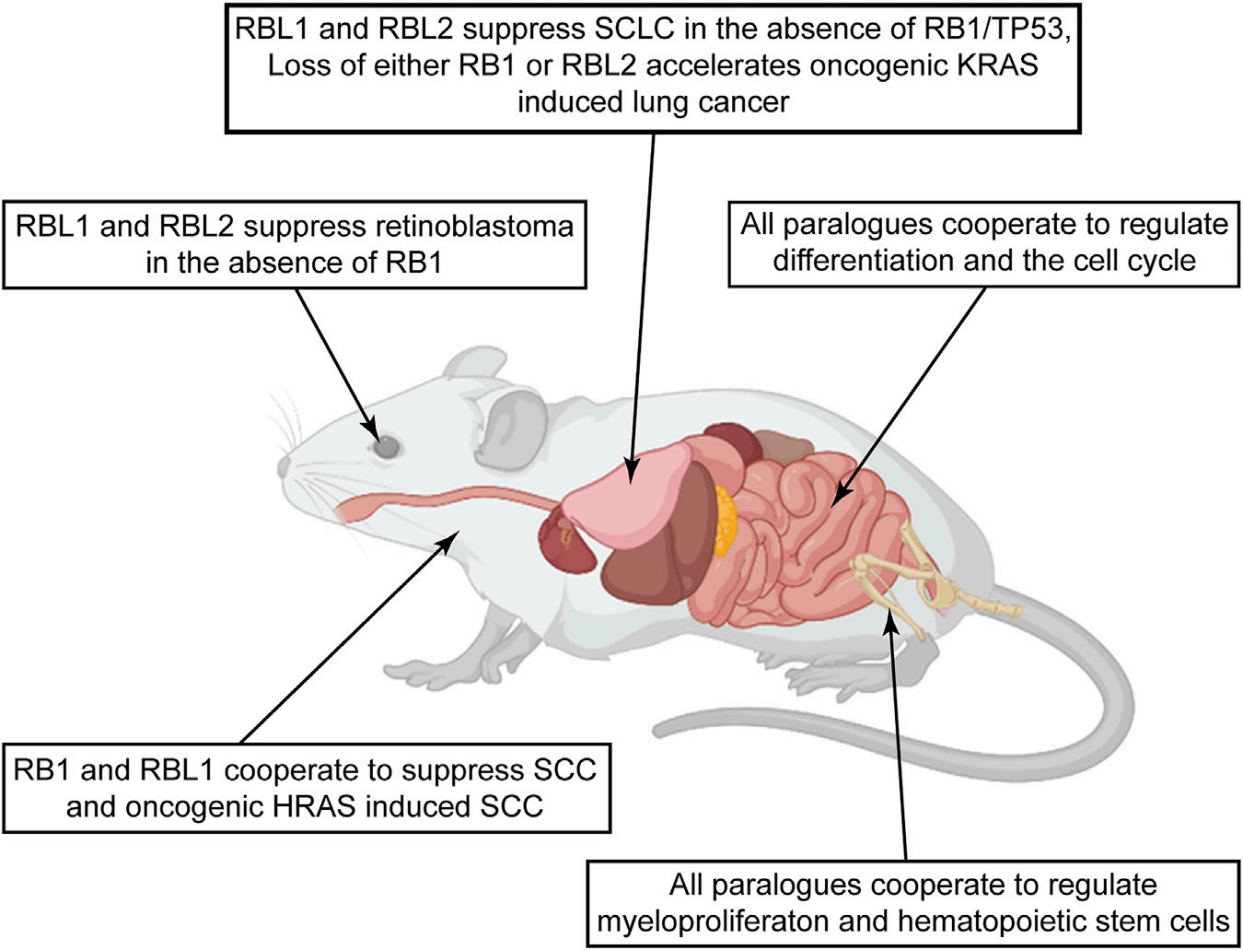
Pocket protein paralogues display diverse tumor suppressor activities across mouse tissues. The figure summarizes studies comparing potential tumor suppressor activities of the pocket protein paralogues in genetically engineered mice. Observations in different tissues highlight evidence of diverse tumor suppressor functions relevant to early tumor initiation or later tumor progression. Of notable interest are examples in the lung and epidermis studies, where the pocket protein paralogues curb specific oncogenic signaling. Created with BioRender.com.

Perhaps the most rigorous means to experimentally assess tumor suppressor functional activity is to evaluate effects of specific genetic mutations on spontaneous cancer initiation and progression in mice. Below we will review examples of such studies that yield more definitive insight on whether debilitating pocket protein gene mutations can drive tumor development, with a focus on experiments in which the effects of pocket protein paralogues have been compared (Figure 5).

### Pocket Protein Tumor Suppressor Activity in the Retina

Experimental evidence implicating *RBL1* and *RBL2* in tumor suppression has roots in attempts to model retinoblastoma in mice. In contrast to humans, loss of *Rb1* is insufficient to cause murine retinoblastoma. However, ablation of *Rb1* plus one additional pocket protein paralogue is sufficient (Chen et al., 2004; Dannenberg et al., 2004; Zhang et al., 2004). The phenotype of resulting retinoblastoma tumors are different depending on the combination of pocket protein paralogues deleted. Ablation of *Rb1*/*Rbl2* leads to rapid development of multifocal retinoblastoma while ablation of *Rb1*/*Rbl1* causes more delayed development of unifocal retinoblastoma. The number and type of somatic copy number alterations spontaneously acquired during retinoblastoma development suggest *Rb1*/*Rbl2* deficient murine retinoblastomas are more like their human counterparts (Kooi et al., 2017). The finding that *Rb1* loss is not sufficient for retinoblastoma in mice is unexpected but has inspired more careful analysis to uncover possible explanations. Donovan et al. (2006) found that the pattern of pocket protein paralogue expression is different in mice and humans. In humans, *RB1* is the primary paralogue expressed throughout retinal development. In mice, however, embryonic retinal progenitor cells express p107 while postnatal retinal progenitor cells express pRb. Both pRb and p130 are expressed in postmitotic retinal cells. Further, there is regulatory compensation between paralogues during retinal development. If pRb expression is abrogated, p107 expression is upregulated. A complementary compensatory mechanism is observed if p107 expression is eliminated causing up regulation of pRb expression. This compensation is not apparent in humans as ablation of pRb in the human fetal retina failed to elicit up regulation of p107. Murine retinoblastoma thus requires loss of multiple pocket proteins because of functional redundancy and feedback regulation between paralogues. This is one of the first formal demonstrations that pocket protein paralogues have overlapping tumor suppressor activity.

### Pocket Protein Tumor Suppressor Activity in the Lung

Pocket protein pathway gene alterations are common and predict poor outcomes in human lung cancer (Bhateja et al., 2019), thus effects of pocket protein gene deletions have been investigated in the lungs of genetically engineered mice. Deletion of *Rb1* in surfactant protein C expressing alveolar type 2 cells causes lung hyperplasia and defects in epithelial differentiation, but not cancer (Simpson et al., 2009). As in the retina, pRb loss was associated with compensatory p107 expression while p130 expression remain unchanged. Deletion of both *Rb1*/*Rbl1*, but not *Rb1*/*Rbl2*, caused development of non-small cell lung cancers in mice after long latency. Widespread deletion of all three pocket protein genes across multiple cell types within the mouse lung induces relatively benign tumorlets that stain positive for neuroendocrine lineage markers (Lázaro et al., 2017). Moreover, these mice are susceptible to chemical carcinogenesis as exposure to DHPN causes development of low grade malignant neuroendocrine tumors while DHPN did not cause tumorigenesis in control mice. These observations suggest all three pocket proteins may influence the initiation of neuroendocrine cancers in the lung. Yet it is *RB1* loss that is a nearly universal feature of human high grade neuroendocrine lung cancers like small cell lung cancer. The other pocket protein genes are rarely altered in these cancers. This emphasizes *RB1*’s unique tumor suppressor activity in the lung.

In human small cell lung cancer *RB1* loss nearly always occurs in combination with genetic alterations in other known oncogenes and tumor suppressor genes, particularly *TP53*. *Rb1* and *Trp53* deletion in the mouse lung causes development of tumors highly reminiscent of human small cell lung cancer with relatively long latency (Meuwissen et al., 2003). The cell type in which these deletions are made has a significant impact on resulting cancer phenotype (Sutherland et al., 2011). Deletion of *Rb1*/*Trp53* in pulmonary neuroendocrine cells efficiently induces small cell lung cancer. Loss of *Rb1*/*Trp53* in alveolar type 2 cells can also drive development of small cell lung cancer, but with reduced penetrance compared to deletion in pulmonary neuroendocrine cells. Clara cells are largely resistant to effects of *Rb1*/*Trp53* deletion. These observations indicate that effects of *Rb1/Trp53* loss are dependent on cell type, efficiently triggering neoplastic transform in some cell types but not triggering neoplastic transformation in others.

The efficiency of transformation also depends on other pocket proteins. Deletion of *Rb1*/*Trp53* plus *Rbl2* greatly accelerates the genesis of small cell lung cancers, and the gene expression pattern of these tumors is similar to the human disease (Schaffer et al., 2010). More recently Ng et al. used CRISPR-Cas9 mediated gene mutation to directly compare effects of p107 and p130 loss on small cell lung cancer induced by *Rb1*/*Trp53* deletion. The ablation of p107 or p130 in addition to *Rb1*/*Trp53* accelerated the rate of tumor progression and decreased the median survival of mice. Interestingly, distinct tumor phenotypes are observed depending on the pocket protein orthologues deleted. *Rb1/Trp53/Rbl1* ablated mice developed fewer but larger tumors compared to *Rb1/Trp53/Rbl2* deleted mice. The *Rbl1*-deleted small cell lung cancers exhibited a higher rate of mediastinal lymph node metastasis and lymphocyte infiltration compared to *Rbl2*-ablated mice. Thus *Rbl1* and *Rbl2* have tumor suppressor activity in the context of small cell lung cancer, but it is only revealed in the absence of *RB1*.

Pocket proteins also influence lung cancers initiated by other oncogenic drivers. *EGFR* and *KRAS* are the most frequently mutated oncogenes in human lung adenocarcinoma. Mutation of these oncogenes also induce lung adenocarcinoma in mice (Fisher et al., 2001; Jackson et al., 2001; Ji et al., 2006; Politi et al., 2006). Foggetti et al. used a CRISPR-Cas9 based approach to screen the effects of select mutations on development of lung adenocarcinoma initiated by *Egfr/Trp53* or *Kras/Trp53* mutations (Foggetti et al., 2021). Loss of *Rb1*, but not *Cdkn2A*, accelerated lung cancer progression induced by *Egfr/Trp53* mutation. Loss of either *Rb1* or *Cdkn2A* accelerated lung cancer progression initiated by *Kras/Trp53* mutation, consistent with the higher frequency of *CDKN2A* alteration in *KRAS* versus *EGFR* initiated human lung adenocarcinoma. Effects of other pocket proteins were not tested. Such observations highlight that the effects of pocket protein pathway genetic alterations also depend on genetic background.

Ho et al. (2009) compared the effects of *Rb1* or *Rbl2* loss on Kras initiated lung adenocarcinoma. The median life span of these mice was reduced by additional loss of either *Rb1* (20 weeks) or *Rbl2* (25 weeks) compared to oncogenic *Kras* alone (32 weeks). However, loss of the pocket protein paralogues accelerated tumor progression differently. Loss of *Rb1* accelerated the early stages of tumor development as indicated by an increased number of adenomas and tumor burden in younger mice, and it increased tumor grade. In contrast, loss of *Rbl2* led to larger tumors in older mice. Analogous to the retina, lung tumors lacking pRb exhibited compensatory upregulation of p130 and p107 expression. Up regulation of p107 expression was also observed when *Rbl2* was deleted in *Kras* mutant lung cancer. This is one of the few examples were a pocket protein paralogue exhibits tumor suppressor activity in the presence of wild type *Rb1*, suggesting *Rb1* cannot completely compensate for loss of *Rbl2* in some contexts.

### Pocket Protein Tumor Suppressor Activity in the Epidermis

The pocket protein pathway is frequently mutated in human skin cancers. *CDKN2A*, for example, is lost in 35% of squamous cell carcinomas. Thus, several studies have focused on pocket protein function in the epidermis. Paramio et al. (1998) find that basal and lower spinous cells of the skin express both pRb and p107 while upper spinous and granular cells express p130. Forced overexpression of pRb and p107 in the human keratinocyte cell line HaCaT induces expression of early differentiation markers, while overexpression of all the pocket proteins induces expression of later differentiation markers. Overexpression of individual pocket proteins inhibit HaCaT cell proliferation, each to a different extent. p107 expression has the strongest inhibitory effect on cell proliferation, followed by pRb and finally p130. In this biological context, all the pocket protein paralogues cooperate to regulate differentiation and proliferation in epidermal cells, cells that exhibit a high turnover rate in order to maintain skin barrier function.

Lara et al. (2008a), Lara et al. (2008b) and Ruiz et al. (2004) have extended the work *in vivo* using genetically engineered mice. Mice lacking pRb in the epidermis develop hyperplasia and hyperkeratosis due to abnormal proliferation and differentiation. However spontaneous tumorigenesis does not occur. Additional deletion of one or both p107 alleles exacerbates this phenotype. Complete loss of *Rb1/Rbl1* causes mice to die shortly after birth, before tumorigenesis can be observed. To circumvent this issue, skin grafts from dual *Rb1/Rbl1*-ablated pups have been transplanted into immunodeficient mice. Papillomatous lesions develop spontaneously from these grafts and subsequently progress to squamous cell carcinomas. Skin grafts lacking pRb alone do not develop tumors. The effects of pocket protein loss in the context of *Hras* oncogenic mutation have also been tested. Primary keratinocytes expressing oncogenic mutant *Hras* and lacking both *Rb1* and *Rbl1* develop into large, poorly differentiated tumors upon transplantation into mouse hosts. Mutant *Hras* expressing keratinocytes lacking *Rb1* alone develop tumors with delayed kinetics and a more differentiated phenotype. Molecular analyses has revealed reduced p53-dependent apoptosis in primary keratinocytes lacking both *Rb1* and *Rbl1*, analogous to findings in mouse skin lacking both these pocket protein paralogues (Costa et al., 2012). This effect may contribute to the more aggressive skin tumors developing in mice lacking both paralogues. Thus both *Rb1* and *Rbl1* have detectable tumor suppressor activity in the skin.

### Pocket Protein Tumor Suppressor Activity in the Intestine

Like the skin, the intestinal epithelium is a dynamic tissue exhibiting high cell turnover. Homeostasis of the intestinal epithelium is maintained by a population of constantly proliferating stem cells that give rise to progeny that progressively differentiate into enterocytes and other support cells as they migrate from the intestinal crypts towards the tips of the villi. Given stereotypical and spatially defined cell cycling within this tissue, it is attractive for assessing coordination between differentiation and the cell cycle. Normally, pRb and p130 expression is uniform across both the crypt and villi while p107 is most strongly expressed in proliferating crypt cells (Haigis et al., 2006; Guo et al., 2009). However, pRb is hyperphosphorylated in crypt cells while it is hypophosphorylated in villus cells. Mice lacking pRb in the intestine develop focal hyperplasia, increased proliferation in the crypt, and ectopic cell cycle re-entry within villus enterocytes, as long as the efficiency of gene deletion is sufficiently high. Differentiation is also impaired in the absence of *Rb1* based on expression of differentiation markers. As in other tissues, the expression of p107 increases in both crypts and villi upon pRb loss. However this compensation is not sufficient to prevent intestinal hyperplasia. Deletion of pairs of pocket protein paralogues exacerbate the proliferative and differentiation phenotypes, with loss of *Rb1* plus either of the other paralogues giving the most severe phenotype. These observations suggest *Rb1* can more completely compensate for *Rbbl1* and/or *Rbl2* loss than either of these paralogues can compensate for *Rb1* loss. As in other tissues noted above, therefore, this evidence suggests *Rb1* function in linking cell cycle exit and differentiation in the intestine is relatively unique compared to other pocket protein family members.

### Pocket Protein Tumor Suppressor Activity in Hematopoietic Stem Cells

Maintenance of quiescence in hematopoietic stem cells is necessary to maintain homeostasis in the bone marrow and prevent the formation of blood cancers such as leukemias and lymphomas. A number of studies have demonstrated that *Rb1* deletion causes defects in hematopoiesis including defective maturation of erythrocytes (Macleod, 2008). However, many of these effects appear to be non-cell autonomous, confounding interpretation of observed phenotypes and the direct role of pRb. One exception to this is the documented effects of *Rb1* loss on hematopoietic stem and progenitor cells under stress conditions where a cell intrinsic effect is observed (Daria et al., 2008). In contrast, loss of *Rbl2* does not yield detectable hematopoietic phenotypes (Cobrinik et al., 1996) while *Rbl1* loss causes mild myeloid hyperplasia that is only evident on the Balb/c genetic background (LeCouter et al., 1998). To explore this further, all three pocket protein paralogues have been deleted in hematopoietic cells of the mouse (Viatour et al., 2008; Kim et al., 2017). These triple knockout mice show reduced viability accompanied by myeloproliferation and myeloid cell infiltration in the kidney, liver, lungs, and spleen. Hematopoietic progenitor cells in the bone marrow of these mice showed increased proliferation and a shift towards the myeloid lineage. This is clearly a cell intrinsic defect as transplantation of triple knockout hematopoietic stem cells into wild type mice yield the same phenotype. Interestingly, mice retaining one wild-type allele of *Rbl1* did not show any of the myeloid or hematopoietic progenitor cell phenotypes. Thus a single *Rbl1* allele can fully compensate for loss of other pocket protein genes to maintain normal hematopoietic stem cell function and homeostasis. These findings highlight the important contributions that all pocket proteins make to normal hematopoiesis and may explain why upstream components of the core pocket protein pathway like cyclins, CDKs, and CDKIs are more commonly altered in leukemias and lymphoma than other cancers since this would functionally compromise all pocket protein paralogues simultaneously.

### Pocket Proteins, Pluripotency, and Cancer Lineage Plasticity

While the canonical function of the pocket protein pathway is to regulate the cell division cycle, it has long been recognized that another hallmark feature of the family is its influence on cellular differentiation and development (Calo et al., 2010; Chinnam and Goodrich, 2011; Viatour and Sage, 2011; Julian and Blais, 2015; Dyson, 2016). Loss of pocket protein activity not only deregulates the cell cycle, but also compromises cellular differentiation. These two cellular processes are intimately linked, and the pocket protein pathway helps maintain this link during normal tissue homeostasis. Pocket protein pathway disruption is expected to sever this link, deregulating both differentiation and the cell cycle in emergent neoplastic cells. While loss of pocket protein cell cycle regulatory activity has long been recognized as a major cancer driver, loss of pocket protein mediated functions in cellular differentiation is an increasingly appreciated aspect of pocket protein tumor suppression.

One line of research inspiring this growing appreciation is the discovery of facultative tissue stem cells and experimentally induced pluripotent stem cells. This work has made clear that cellular differentiation is not irreversible and that normal cells can dedifferentiate into a stem or progenitor phenotype. *RB1* is a barrier to this reversibility as loss of pRb increases the efficiency of induced stem cell reprogramming while pRb overexpression reduces reprogramming efficiency (Kareta et al., 2015). In this context, *RB1* status does not alter cell cycle control. Instead pRb directly regulates the expression of pluripotency inducing transcription factors by binding to the relevant genes in complex with E2Fs, recruiting chromatin regulatory complexes like EZH2/PRC2 (Chicas et al., 2010; Kareta et al., 2015), and repressing RNA transcription. Indeed, *RB1* loss can replace the requirement for *SOX2* during induced stem cell reprogramming. These effects are not replicated by manipulation of the other pocket protein paralogues, highlighting a potentially unique aspect of pRb function. However, complete loss of all pocket protein paralogues is lethal for embryonic stem cells (Conklin et al., 2012), suggesting some level of pocket protein activity is needed to maintain the viability of pluripotent cells. This pRb-mediated effect on pluripotency is relevant to cancer as stem cell reprogramming factors like *SOX2* are required for tumorigenesis initiated by *RB1* loss (Li et al., 2012; Vilas et al., 2015). *RBL2* has also been implicated in the direct regulation of *SOX2* expression, but not *RBL1* (Li et al., 2012; Vilas et al., 2015). This suggests *RB1* uniquely regulates other aspects of pluripotency transcriptional networks to account for observed differences in the effects of pocket protein paralogues on induced stem cell reprogramming.

A second line of research emphasizing the importance of pocket protein mediated regulation of differentiation has been in area of cancer lineage plasticity. Cancer lineage plasticity describes the ability of cancer cells to reprogram their lineage state in order to adapt to selective pressures like therapy (Le Magnen et al., 2018; Quintanal-Villalonga et al., 2020). This mechanism of acquired therapeutic resistance has become more apparent in recent years as increasingly potent molecularly targeted therapies have been more widely deployed in the clinic (Sequist et al., 2011; Bluemn et al., 2017). The two key examples discussed above include prostate adenocarcinoma and *EGFR* mutant non-small cell lung cancer, two diseases where standard of care involves highly effective therapies targeting androgen receptor or EGFR signaling, respectively. In both diseases, a significant fraction of patients will relapse through therapy in association with an altered lineage state, including aggressive neuroendocrine variants. These lineage variants typically lack expression of the therapeutic target and are no longer dependent on it for growth and survival. This lineage transformation is associated with loss of *RB1* and *TP53* (Niederst et al., 2015; Ku et al., 2017; Mu et al., 2017), but does not directly involve changes in the cell cycle. Rather it involves epigenetic reprogramming promoted by factors reminiscent of those involved in stem cell reprogramming, like SOX2 and EZH2/PRC2. *RB1*/*TP53* loss is not sufficient for neuroendocrine lineage transformation as many *RB1*/*TP53* deficient cancers do not exhibit an obvious lineage state change. Even without a lineage state change, *RB1*/*TP53* loss confers resistance to a wide range of cancer therapies. Perhaps this is because signals from many molecules targeted by cancer therapy converge on pocket protein pathway activity and depend on activities like pocket protein cell cycle control for therapeutic effectiveness (Knudsen et al., 2019; Nyquist et al., 2020). It is unclear whether loss of other pocket protein paralogues will phenocopy loss of *RB1* in the context of cancer lineage plasticity and therapeutic resistance as systematic comparisons are lacking in the published literature.

## Conclusions and Speculations

Both human cancer genome sequencing data and experimental studies in genetically engineered mice formally demonstrate that all three pocket protein paralogues can function as tumor suppressors, but this data also reinforces the diversity of paralogue tumor suppressor activity and a dominant role for *RB1* (Figure 5). Evidence for *RB1* mediated tumor suppression is more readily detected across a wider spectrum of tissues and biological contexts while evidence for *RBL1* and *RBL2* tumor suppressor activity is more restricted. In many cases the tumor suppressor activity of *RBL1* or *RBL2* is only revealed in the absence of *RB1*. There is less evidence indicating loss of *RBL1* or *RBL2* potentiates cancer initiation or progression in contexts where *RB1* remains intact. Nonetheless this conclusion is consistent with our understanding of pocket protein structure and function where they exhibit partially overlapping similarities, p107 and p130 being more similar to each other than they are to pRb. The greatest overlap between the pocket protein paralogues seems to converge on their cell cycle related functions, although even here there are differences in detail.

The body of data reviewed here also reveals that genetic disruption of pocket protein pathway function has non-uniform effects that differ depending on cancer type and the cancer’s genetic background. This is somewhat surprising given the widely held assumption that enforcing cell cycle control is the primary tumor suppressor function for the pathway. This potential paradox is particularly evident in cancers where genetic alteration of the pathway occurs late in cancer progression, well after the cell cycle is deregulated sufficiently to support development of the primary tumor. This apparent paradox can be resolved through identification of pocket protein mediated, context dependent, and non-canonical functions beyond its more universal role in the cell cycle. Rapidly accumulating experimental data has implicated the pocket protein pathway in multiple functions beyond canonical cell cycle control. A systematic review of all these functions is beyond the scope of this effort, but the topic has been addressed recently elsewhere (Dick et al., 2018; Knudsen et al., 2019). These non-canonical functions are likely to account for both the context dependent effects of pocket protein pathway disruption and the unique role for pRb in tumor suppression.

The cellular and molecular mechanisms mediating pRb’s unique tumor suppressor activity are not well understood. Nonetheless, we can speculate based on known differences between pocket protein paralogues from which these unique mechanisms may arise. The most fundamental differences are in their structure and biochemical activity. The pocket proteins all function as protein adaptors linking sequence specific DNA binding factors with protein complexes that modify chromatin. However, structural differences in their pocket domains specify preferential interactions with overlapping but distinct subsets of cellular proteins. Differences in the pocket protein interacting proteomes can potentially specify qualitatively distinct functions. *RB1* has been implicated uniquely in some cancer relevant cellular functions, including pluripotency and cancer lineage plasticity. We propose that this pRb-specific function is a good candidate mechanism accounting for *RB1*’s dominant role in tumor suppression. *RB1* not only derepresses pluripotency transcriptional networks, but loss of cell cycle control likely exacerbates epigenetic instability by destabilizing chromatin during cell cycle coupled disassembly and re-assembly. Resulting *RB1* loss driven cancer lineage plasticity may facilitate adaptation of cancer cells to selective pressures experienced during metastatic dissemination and therapy, thereby driving cancer progression.

It is also clear that the pocket proteins are expressed in different cell types and in different stages of the cell cycle. This may also contribute to pRb’s unique tumor suppressor role if the cells where it normally functions are more likely to serve as the cells of cancer origin. *RBL2* is expressed most highly in non-proliferative cells while *RBL1* is expressed primarily in S phase. *RB1* expression bridges these two and thus may be important in cell types transitioning between proliferative and non-proliferative states, like progenitor cells undergoing terminal differentiation. Or, pRb loss may have unique effects in pre-existing cancer cells. Loss of p107 or p130 may have little effect on pre-existing cancer cells while pRb loss may have more robust effects, such as increasing cancer lineage plasticity. Thus the unique role for pRb in cancer may be a function of both the unique molecular mechanisms it mediates and the unique cell types/states in which it carries out these functions.

An important caveat with the central conclusion of this review is that experimental studies systematically comparing the tumor suppressor activity of pocket protein paralogues are not abundant. Most studies focus on *RB1* because of its already established importance in cancer, but this only further reinforces the bias. If we are to better understand the diversity of pocket protein tumor suppressor activity, the molecular mechanisms accounting for this diversity, and the clinical ramifications of these divergent mechanisms, future studies will need to address this bias by comparing pocket protein tumor suppressor activity systematically and directly.
